# The impact of technology transfer on the green innovation efficiency of Chinese high-tech industry

**DOI:** 10.3389/fsoc.2023.1141616

**Published:** 2023-07-17

**Authors:** Shuzhen Zhou, Feng Peng

**Affiliations:** ^1^China Three Gorges University Library, China Three Gorges University, Yichang, China; ^2^Research Center for China Industry-University-Research Institute Collaboration of Wuhan University, Wuhan University, Wuhan, China

**Keywords:** green innovation efficiency, technology transfer, environmental pollution, high-tech industry, three-stage network data envelopment analysis

## Abstract

Promoting technology transfer is an important strategic measure for China to promote industrial innovation. However, there is little research exploring the influence of technology transfer on the green innovation efficiency (GIE) of China's high-tech industry (HTI). From the perspective of process, green innovation in HTI is a continuous three-stage system including research and development (R&D), commercialization, and diffusion. Therefore, we measure the GIE of China's HTI by using a three-stage network data envelopment analysis (NDEA) model considering environmental pollution and establish a series of regression models to investigate the role of the two main ways of technology transfer, domestic technology acquisition (DTA) and foreign technology introduction (FTI), in improving the GIE of HTI. The results show that the average GIE of China's HTI is 0.7727 from 2011 to 2020. Except for Jiangsu, Guangdong, Qinghai, and Xinjiang, green innovation in HTI in other provinces in China is inefficient. DTA has significantly promoted GIE in HTI. FTI has a positive impact on the GIE of HTI but is not statistically significant. The robustness test confirmed these results. This study is helpful to understand the differences between the effects of DTA and FTI on the GIE of China's HTI, to provide a basis for adjusting technology transfer policies.

## 1. Introduction

**Figure 1:**
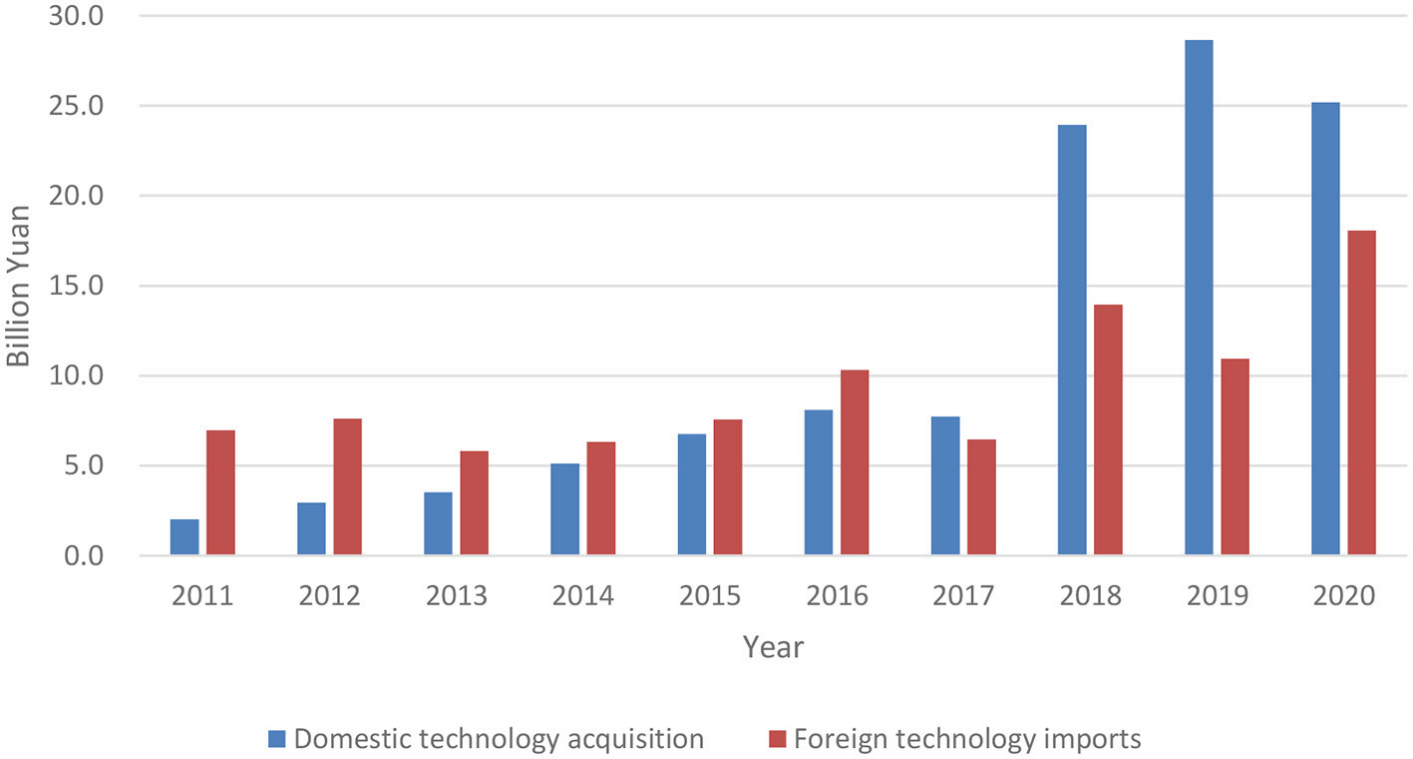
Expenditure of two types of technology transfer from 2011 to 2020.

High-tech industry (HTI) refers to technology-intensive industries with high research and development (R&D) intensity and high product added value. It is characterized by innovation and environmental friendliness. It plays an important role in enhancing the competitiveness of the manufacturing industry and promoting economic structural optimization, making it a crucial area in international competition. Technology transfer is an important pathway to promote green development in industries (Fernandes et al., 2021; Zheng et al., 2022). China has actively pursued practical exploration of technology transfer. In September 2017, the State Council of China issued the National Technology Transfer System Construction Plan, with a view toward using technology transfer to provide support for improving the capability of green innovation. According to data released by the National Bureau of Statistics of China, the domestic technology acquisition (DTA) expenditure of Chinese HTI increased from 2.0241 billion yuan in 2011 to 25.1917 billion yuan in 2020. Foreign technology introduction (FTI) funds decreased, however, from 6.9650 billion yuan in 2011 to 18.0730 billion yuan in 2020 (as shown in Figure 1). Facing the dual constraints of limited innovation resources and deteriorating ecological environment (Peng et al., 2022), it is essential to examine the relationship between technology transfer and green innovation capability from the perspective of process to promote the sustainable development of Chinese HTI. Relevant empirical studies, however, remain lacking.

Some studies have evaluated the green innovation efficiency (GIE) of Chinese HTI. Research to date can be divided into two categories: (1) the “black-box” perspective, which regards the green innovation of HTI as a “black-box” and evaluates the input-output conversion efficiency of this “black-box” (Li L. et al., 2018; Luo et al., 2019); and (2) the process perspective, which regards the green innovation of HTI as a multistage continuous process and evaluates the input-output efficiency of each stage (Deng et al., 2020). Compared with the “black box” perspective, the process perspective can further our understanding of industrial GIE and its components.

Some research has analyzed the impact of technology transfer on GIE in HTI from the “black box” perspective. Liu et al. (2020) found that in the areas with strong competitiveness of HTI, DTA was significantly positively related to the GIE of HTI, whereas the role of FTI was just the opposite. However, few studies have examined the differences between the two ways of technology transfer in improving the efficiency of green innovation from a process perspective (Liu et al., 2020). In addition, when measuring GIE in HTI, existing studies often choose sulfur dioxide (SO2) emissions as a single undesirable output indicator, which may lead to measurement bias of the results of GIE (Yang et al., 2020).

There are two main contributions in this paper. First, from the perspective of process, we decomposed the green innovation process of HTI into three stages: R&D, commercialization, and diffusion. On this basis, a three-stage index system of green innovation in HTI was constructed, and the network data envelopment analysis (NDEA) model considering environmental pollution was used to measure the GIE of inter-provincial HTI in China. Second, after measuring the GIE, a series of regression models are constructed to examine the differences between the two ways of technology transfer, DTA and FTI, in promoting the GIE of HTI.

In Section 2, we review the theoretical literature on the impact of technology transfer on GIE in HTI. In Section 3, we introduce our research methods, including the three-stage NDEA model and the regression model. In Section 4, we provide estimated results of the impact of technology transfer on industrial GIE. In Section 5, we render conclusions and limitations.

## 2. Theoretical background

The use of technology transfer can help enterprises overcome internal constraints that affect their green development, such as lack of capacity or input (Ghisetti et al., 2015). Technology transfer plays a crucial role in facilitating green innovation (Leiponen and Helfat, 2010; Hu et al., 2017). DTA and FTI are the two main types of technology transfer (Li et al., 2020; Qian et al., 2022).

The use of technology transfer can help enterprises overcome internal constraints that affect their green development, such as the lack of capacity or input (Ghisetti et al., 2015). Technology transfer plays a crucial role in facilitating green innovation (Leiponen and Helfat, 2010; Hu et al., 2017). DTA and FTI are the two main types of technology transfer (Li et al., 2020; Qian et al., 2022).

DTA is an important channel to obtain external technology. Enterprises can obtain the technical knowledge needed for their product or process innovation from local universities and research institutions to supplement or replace expensive R&D activities (Caloghirou et al., 2004). Because the technology gap of these domestic enterprises is relatively small, recipients can better digest, and absorb domestic technology (Deng and Lu, 2021). Furthermore, the same knowledge background of domestic enterprises can reduce transaction costs and information asymmetry (Li, 2011). Some high-tech enterprises improve the efficiency of innovation through industry-university-research cooperation (Chen et al., 2016). It is often difficult for developing countries to acquire specialized, diversified, and advanced technical knowledge when acquiring domestic technologies, however, and it may be difficult to help them accelerate their innovation process (Elia et al., 2020).

FTI is another important channel of technology transfer. Latecomer countries can carry out technological innovation based on introduced technology and catch up technologically in a short time (Awate et al., 2015; Yu et al., 2019). The introduction of high technology will enable developing countries to obtain technology spillover effects (Belitz and Molders, 2016). Nevertheless, technology introduction may cause developing countries to fall into the “technology dependence trap,” which can lead to the inhibition of their independent innovation capacity (Laursen and Salter, 2006; Choi, 2017). In addition, the introduced technology may contain highly polluting mechanical equipment, which may cause damage to the environment (Peters and Hertwich, 2006; Tukker et al., 2013). Therefore, the influence of FTI on GIE is uncertain.

Green innovation requires enterprises to deal with complex technological and economic problems, therefore, requires knowledge input from different technological sources (Cainelli et al., 2015; Ketata et al., 2015). The effectiveness of green innovation is influenced by the source of technical knowledge, but most importantly, by the combination of technical knowledge in the green innovation process (Ben Arfi et al., 2018). Therefore, the process perspective will provide a new understanding of the differences in the role of different technology transfer modes in the improvement of GIE in HTI.

## 3. Methodology

### 3.1. Model

To analyze the influence of technology transfer on the GIE of Chinese HTI, we set the following model:


(1) $$\begin{array}{l} {GIE}_{it}\;=\;\alpha_{0}\;+\;\alpha_{1}D{TA}_{it}\;+\;\alpha_{2}{FTI}_{it}\;+\;\gamma_{k}\emptyset_{it}\;+\;\varepsilon_{it} \end{array}$$

where the subscripts *i* and *t* represent the province and year, respectively; *GIE* represents the GIE of HTEs; *DTA* represents the domestic technology acquisition; *FTI* represents the foreign technology introduction; ∅ is the control variable vector; and ε represents the random error.

### 3.2. Dependent variable

From the perspective of process, the green innovation process of HTI can be divided into three stages: R&D, commercialization, and diffusion (Lin et al., 2023). The input-output variables of these three stages are shown in Table 1.

**Table 1:** Input-output variables in the process of three-stage green innovation.

**Phase**	**Category**	**Item**	**Variable**
R&D phase	Input	Original input	R&D expenditure
			R&D full-time equivalent
	Output	Intermediate	Number of patent applications
			Number of patents in force
Commercialization phase	Input	Intermediate	Number of patent applications
			Number of patents in force
		Additional input	New product development expenditure
	Output	Intermediate	Sales income of new products
Diffusion phase	Input	Intermediate	Sales income of new products
		Additional input	Number of new product development projects
	Output	Desirable output	Revenue from main business
		Undesirable output	SO_2_ emissions
			Solid waste emissions

The input in the R&D stage is R&D full-time equivalent and R&D expenditure (Wang et al., 2016; Du et al., 2019). Its output is patent applications and patents in force (Zhang et al., 2019). These outputs are also inputs in the commercialization phase. Supplementary input in the commercialization stage is the expenditure for new product development (Du et al., 2019). The output is the sales income of new products. This output is also the input of the diffusion phase. The supplementary input in the diffusion stage is the number of new product development projects (Chen et al., 2021b). The desirable output of the diffusion stage is the main business income (Lin et al., 2023), and its undesirable output is environmental pollution emissions. Due to the availability of data, SO2 emissions and wastewater emissions were selected as undesirable outputs in this paper (Yang et al., 2020; Chen et al., 2021a).

Stochastic frontier analysis (SFA) and DEA are common methods used to measure GIE. The SFA method offers advantages in dealing with measurement error and statistical interference (Zhu et al., 2021), but it is difficult to use when dealing with the input-output efficiency evaluation of multiple stages and multiple outputs (Li T. et al., 2018). The DEA method is often used to measure the relative efficiency of the same kind of decision-making units (DMUs) with multiple inputs and outputs (Tang and Qin, 2021), and it can provide improvement basis for increasing desirable outputs and reducing undesirable outputs of non-effective DMUs (Liu et al., 2020).

From the process perspective, the process of green innovation in HTI includes multiple stages and involves a variety of inputs and outputs. Therefore, the network DEA model is needed to measure GIE (Cook et al., 2010; Du et al., 2019).

Assume that $x_{ij}^{l}$ and $y_{rj}^{l}$ represent the *i*th input and the *r*th output of the *j*th DMU at the *l*th node (phase), respectively; $z_{f_{\left(l,l^{{}^{\prime }}\right)j}}^{\left(l,l^{{}^{\prime }}\right)}$ represents the intermediate output of the *j*th DMU between the *l*th node (phase) and the *l*′th node (phase); and the subscript $f_{\left(l,l^{{}^{\prime }}\right)}$ indicates the number of intermediate outputs between the *l*th node (phase) and the *l*′th node (phase), $f_{\left(l,l^{{}^{\prime }}\right)}=1,\;\cdots \;,\;F_{\left(l,l^{{}^{\prime }}\right)}$. The NDEA model can be expressed as follows (Tavana et al., 2013):


(2) $$\gamma^{*}\;=\;\min\;\sum_{\begin{array}{c} l=1\\ \theta_{k\;},\;\lambda ,\;S^{-} \end{array}}^{k}W_{l}\left(\theta_{l}-\varepsilon_{x}^{l}\sum_{i=1}^{m_{l}}\frac{w_{i}^{l-}S_{i}^{l-}}{x_{i_{0}}^{l}}\right)$$

$$\begin{array}{l} s.t.\\ \sum_{j=1}^{n}x_{ij}^{l}\lambda_{j}^{l}+S_{i}^{l-}=\;\theta_{h}x_{i0}^{l},\\ \sum_{j=1}^{n}y_{rj}^{l}\lambda_{j}^{l}\geq y_{r0}^{l},\\ \sum_{j=1}^{n}z_{f_{\left(l,l^{{}^{\prime }}\right)j}}^{\left(l,l^{{}^{\prime }}\right)}\lambda_{j}^{l}\;=\;\sum_{j=1}^{n}z_{f_{\left(l,l^{{}^{\prime }}\right)j}}^{\left(l,l^{{}^{\prime }}\right)}\lambda_{j}^{l^{{}^{\prime }}},\\ i\;=\;1,\;\cdots ,\;m_{l},\;l\;=\;1,\;\cdots ,\;k,\\ r\;=\;1,\;\cdots ,\;s_{l},\;l\;=\;1,\;\cdots ,\;k,\\ f_{\left(l,l^{{}^{\prime }}\right)}\;=\;1,\;\cdots ,\;F_{\left(l,l^{{}^{\prime }}\right)},\;\forall \left(l,l^{{}^{\prime }}\right),\\ \theta_{l}\leq 1,\;l\;=\;1,\;\cdots ,\;k,\\ \lambda_{j}^{l}\geq 0,\;j\;=\;1,\;\cdots ,\;n,\;l\;=\;1,\;\cdots ,\;k,\\ s_{i}^{l-}\geq 0,\;i\;=\;1,\;\cdots ,\;m_{l},\;l\;=\;1,\;\cdots ,\;k, \end{array}$$

where $w_{i}^{l-}$ represents the weight of the *i*th input of the *l*th node (phase), which satisfies $\overset{m_{l}}{\underset{i=1}{\sum }}w_{i}^{l-}=1$; $\varepsilon_{x}^{l}$ is used to measure the dispersion of various inputs of the *l*th node (phase); $\varepsilon_{x}^{l}$ represents the relaxation of the *i*th input of the *l*th node (phase); and *W*_*l*_ represents the weight of the *l*th node (phase).

### 3.3. Explanatory variables and control variables

#### 3.3.1. Explanatory variables

Explanatory variables include DTA and FTI. DTA is measured by the ratio of the expenditure on the purchase of domestic technology to the main business income of HTI. FTI is measured by the ratio of expenditure on the introduction of foreign technology to the main business income of HTI.

#### 3.3.2. Control variables

In addition to these two types of technology transfer, the existing literature also has identified other influencing factors of GIE in HTI, including enterprise scale (ES), marketization level (ML), foreign direct investment (FDI), human capital (HC) level, and environmental regulation (ER) (Li L. et al., 2018; Li T. et al., 2018; Peng et al., 2018; Zhang et al., 2022).

(1) ES

Green innovation is costly and risky. Large-scale enterprises have more abundant resources for green innovation, and thus, they are more able to bear the costs and risks of green innovation (Li T. et al., 2018). As the enterprise grows in scale, however, its innovation management efficiency also may decrease (Zhu et al., 2021).

(2) ML

The cooperation between technology suppliers and consumers has an important impact on improving the utilization rate of technology (Li L. et al., 2018). The market is a platform for technology transfer and diffusion. A mature market can enhance the cooperation between technology suppliers and demanders, thus promoting the transfer and diffusion of technology more effectively (Li T. et al., 2018).

(3) FDI

FDI from developed countries usually has technology spillover effect on enterprises in developing countries (Sari et al., 2016; Vujanovic et al., 2022). This provides a technological basis for enterprises in developing countries to achieve green innovation (Feng et al., 2018; Luo et al., 2021).

(4) HC

The HC level affects firms' ability to absorb external technologies (Kneller and Stevens, 2006; Huang et al., 2019). Firms with higher HC levels are better able to adopt external technologies than others (Blalock and Gertler, 2009; Guo et al., 2022).

(5) ER

ER increases the expenditure of controlling environmental pollution for enterprises and squeezes out the funds for technological innovation (Zhang et al., 2021). ER, however, also can encourage enterprises to carry out technological innovation, which may introduce more benefits (Li and Zeng, 2020).

These factors are widely used in the empirical study of GIE in Chinese HTI. Zhang et al. (2022) confirmed that ES is significantly positively correlated with industrial GIE. Li T. et al. (2018) confirmed that ML has a positive impact on GIE. Peng et al. (2018) found a significant positive correlation between FDI and GIE. Yang et al. (2022) found that the HC level has a significant positive impact on GIE. Li L. et al. (2018) found that ER has a significant negative impact on GIE. Therefore, we chose FDI, HC level, ML, ER, and ES as control variables.

FDI is expressed as a ratio of the number of foreign-funded enterprises in HTI (Xu et al., 2020). ML is expressed by the ratio of non-state-owned enterprises in the main business income of this (Wang et al., 2021). HC level is expressed by the proportion of employees in the local population (Wang and Zhao, 2021). Per capita GDP is used as the proxy variable for ER (Antweiler et al., 2001). ES is expressed by the average value of the main business income of enterprises (Li T. et al., 2018).

The data used to calculate SO2 emissions and solid waste emissions come from the China Environmental Statistics Yearbook 2012–2021. Data used to calculate environmental regulation come from the China Statistical Yearbook 2012–2021. The data used to calculate other variables come from the Statistical Yearbook of China's High-tech Industry 2012–2021.

The descriptive statistical results of the variables are shown in Table 2. Considering the integrity of the data, we selected the panel data of 30 provinces in China from 2011 to 2020 to examine the impact of technology transfer on the GIE of China's HTI.

**Table 2:** Descriptive statistical results.

**Variable**	**Mean**	**Std. Dev**.	**Min**	**Max**	**Observations**
GIE	0.7727	0.1601	0.3942	1.0000	300
DTA	0.0854	0.3819	0.0000	5.7734	300
FTI	0.0337	0.0623	0.0000	0.5273	300
ES	0.3955	0.1670	0.0676	0.7961	300
ML	83.0340	13.3334	34.3984	100.0000	300
FDI	9.8640	10.0181	0.0000	53.5211	300
HC	0.7547	0.8235	0.0172	3.6267	300
ER	5.6386	2.7307	1.6413	16.4889	300

## 4. Results and discussion

### 4.1. Measurement of GIE

Based on the panel data of 30 provinces in China from 2011 to 2020, we use equation (2) to calculate the GIE of China's HTI (see Table 3).

**Table 3:** GIE in Chinese HTI.

**Province**	**2011**	**2012**	**2013**	**2014**	**2015**	**2016**	**2017**	**2018**	**2019**	**2020**	**Mean**
Beijing	0.6728	0.6906	0.6548	0.6635	0.6206	0.7345	0.7412	0.7176	0.7530	0.7175	0.6966
Tianjin	0.6792	0.7137	0.7283	0.6720	0.6633	0.7112	0.6865	0.6370	0.6484	0.6130	0.6753
Hebei	0.6060	0.6516	0.6201	0.6023	0.6121	0.7261	0.7096	0.6700	0.7164	0.6104	0.6525
Liaoning	0.6724	0.6890	0.6726	0.6949	0.6335	0.6630	0.6680	0.6525	0.6884	0.6434	0.6677
Shanghai	0.9945	1.0000	1.0000	0.9020	0.7685	0.8593	1.0000	1.0000	1.0000	1.0000	0.9524
Jiangsu	1.0000	1.0000	1.0000	1.0000	1.0000	1.0000	1.0000	1.0000	1.0000	1.0000	1.0000
Zhejiang	0.6565	0.6906	0.6611	0.6825	0.6843	0.7562	0.7661	0.7355	0.7730	0.7280	0.7134
Fujian	1.0000	0.8790	0.9158	0.9036	0.8136	0.8841	0.9578	0.9066	0.9583	0.8595	0.9078
Shandong	0.7047	0.7339	0.7300	0.7277	0.6771	0.7418	0.7373	0.7121	0.7476	0.6647	0.7177
Guangdong	1.0000	1.0000	1.0000	1.0000	1.0000	1.0000	1.0000	1.0000	1.0000	1.0000	1.0000
Hainan	0.5032	0.6299	0.5680	0.5733	0.5135	0.7012	0.6880	0.6728	0.7274	0.5637	0.6141
Shanxi	0.7045	0.7559	0.7708	1.0000	0.8495	0.9260	0.8851	0.8471	0.8735	0.7907	0.8403
Jilin	0.6409	0.6853	0.5435	0.7022	0.6652	0.7421	0.7120	0.6522	0.7060	0.5474	0.6597
Heilongjiang	0.4521	0.5104	0.4771	0.5141	0.4524	0.6880	0.5422	0.4858	0.5215	0.4093	0.5053
Anhui	0.5683	0.6703	0.6567	0.7026	0.6963	0.7675	0.7744	0.7669	0.8178	0.7504	0.7171
Jiangxi	0.6514	0.7148	0.6815	0.7759	0.7184	0.7713	0.7787	0.7673	0.8018	0.7442	0.7405
Henan	0.6941	1.0000	1.0000	1.0000	1.0000	1.0000	1.0000	1.0000	1.0000	1.0000	0.9694
Hubei	0.6045	0.6800	0.6816	0.6768	0.6790	0.7605	0.7689	0.7423	0.7792	0.7112	0.7084
Hunan	0.6587	0.7714	0.7321	0.7103	0.6974	0.7766	0.7702	0.7324	0.7772	0.7072	0.7334
Inner Mongolia	1.0000	1.0000	1.0000	1.0000	0.7230	0.7567	0.7676	0.7805	0.8140	0.7080	0.8550
Guangxi	0.6401	0.7537	0.7919	1.0000	1.0000	1.0000	1.0000	1.0000	1.0000	0.8911	0.9077
Chongqing	0.7209	0.8759	1.0000	0.9217	0.8966	1.0000	1.0000	1.0000	1.0000	1.0000	0.9415
Sichuan	0.6491	0.6978	0.6838	0.6837	0.7069	0.8197	0.8063	0.7687	0.8117	0.7878	0.7416
Guizhou	0.3942	0.4351	0.4270	0.5380	0.5403	0.6872	0.7018	0.6946	0.7266	0.6355	0.5780
Yunnan	0.4972	0.6183	0.6300	0.6083	0.5200	0.7102	0.7185	0.7138	0.7796	0.7434	0.6539
Shaanxi	0.4963	0.5048	0.4949	0.5397	0.6045	0.7028	0.7095	0.6859	0.7107	0.6865	0.6136
Gansu	0.4665	0.5734	0.5760	0.5811	0.5827	0.7217	0.7198	0.7097	0.7603	0.7061	0.6397
Qinghai	1.0000	1.0000	1.0000	1.0000	1.0000	1.0000	1.0000	1.0000	1.0000	1.0000	1.0000
Ningxia	0.5296	0.7320	0.6845	0.7583	0.9087	0.8871	0.8563	0.7902	0.8286	0.7956	0.7771
Xinjiang	1.0000	1.0000	1.0000	1.0000	1.0000	1.0000	1.0000	1.0000	1.0000	1.0000	1.0000

As shown in Table 3, Jiangsu, Guangdong, Qinghai, and Xinjiang are the provinces with high GIE in China's HTI, and the green innovations of these four provinces are all effective. Except for these four provinces, green innovation in HTI in other provinces is ineffective. Among them, Heilongjiang has the lowest green innovation efficiency, with an efficiency value of only 0.5053. The average GIE of China's HTI is 0.7727.

### 4.2. Regression results

The value of GIE calculated by the NDEA model is between 0 and 1. For restricted dependent variables, the use of OLS regression can lead to inconsistent estimates. Tobit regression is a common method to analyse this type of sample data (Chen, 2014). Therefore, we use the Tobit model to analyse the impact of technology transfer on the GIE of China's HTI.

Equation (1) is used to analyse the influence of technology transfer on GIE in HTI. Model 1, Model 2, and Model 3, respectively, introduced a series of control variables by stepwise regression method. These control variables include ES, ML, FDI, HC, and ER. The final estimated results are shown in Table 4. The results of the likelihood ratio test (LR test) confirm that the Tobit regression method of random effects should be used for all three models.

**Table 4:** Influence of technology transfer on GIE in Chinese THI.

**Variables**	**(1)**	**(2)**	**(3)**
DTA	0.0231^**^	0.0209^**^	0.0210^**^
	(2.25)	(2.04)	(2.12)
FTI	0.0191	0.0486	0.0692
	(0.24)	(0.59)	(0.86)
ES	0.4076^***^	0.3608^***^	0.3069^***^
	(8.64)	(7.16)	(5.72)
ML	0.0037^***^	0.0034^***^	0.0030^***^
	(6.67)	(6.20)	(5.46)
FDI		−0.0034^***^	−0.0007
		(−2.63)	(−0.44)
HC		0.0578^**^	0.0135
		(2.48)	(0.48)
ER			0.0311^***^
			(3.24)
ER^2^			−0.0013^***^
			(−2.78)
Constant	0.3030^***^	0.3314^***^	0.2691^***^
	(5.95)	(6.46)	(4.91)
Log likelihood	357.5537	361.7736	367.2400
LR test	294.16^***^	270.77^***^	280.48^***^
Observations	300	300	300
Number of province	30	30	30

^***^p < 0.01, ^**^p < 0.05, ^*^p < 0.1.

Models 1, 2, and 3 show that DTA has a significant positive impact on GIE. The coefficients are 0.0231, 0.0209, and 0.0210 respectively. Models 1, 2 and 3 also show that there is a positive correlation between FTI and GIE, with coefficients of 0.0191, 0.0486, and 0.0692 respectively, but it is not statistically significant. The coefficients of ES and ML are significantly positive, indicating that both ES and ML can promote GIE. After considering ER, the impact of FDI and HC on the GIE is no longer significant. The relationship between ER and GIE forms an inverted U. This relationship shows that moderate ER is conducive to green innovation, but that strict ER may be harmful to GIE in HTI.

For comparison, Table 5 shows the estimated results of the fixed-effects model (using Cluster-Robust Standard Errors). It can be found that whether using Tobit random effect model or fixed effect model, the results show that DTA has a significant positive impact on GIE. The FTI is positively related to GIE, but it is not statistically significant.

**Table 5:** Estimated results of fixed effect model.

**Variables**	**(4)**	**(5)**	**(6)**
DTA	0.0213^***^	0.0190^***^	0.0202^***^
	(3.95)	(3.54)	(3.79)
FTI	0.0329	0.0633	0.0932
	(0.40)	(0.64)	(0.78)
ES	0.4274^***^	0.3777^***^	0.3008^***^
	(5.53)	(4.46)	(3.60)
ML	0.0033^***^	0.0031^***^	0.0027^**^
	(3.09)	(2.78)	(2.38)
FDI		−0.0029^*^	0.0033^*^
		(−1.70)	(1.85)
HC		0.0684^*^	−0.0195
		(1.75)	(−0.44)
ER			0.0447^***^
			(2.98)
ER^2^			−0.0017^**^
			(−2.22)
Constant	0.3245^***^	0.3413^***^	0.2251^**^
	(3.76)	(3.81)	(2.36)
Observations	300	300	300
Number of province	30	30	30

^***^p < 0.01, ^**^p < 0.05, ^*^p < 0.1.

### 4.3. Robustness test

We used three methods to test the robustness of the estimates. First, we introduced more control variables into the regression model. Considering the influence of location factors, MID is used to represent the dummy variable of the central region, and WEST is used to represent the dummy variable of the western region. Model 7 shows that although location factors have a significant impact on GIE, the estimation results of independent variables do not change with the addition of more control variables. Second, this paper uses short panel data (N = 30, T = 10). Due to the small-time dimension T, it is difficult to test the hypothesis of autocorrelation and heteroscedasticity. In this case, we use the panel corrected standard error (PCSE) method to give a consistent estimate. Model 8 shows that the estimates are still robust. Finally, the regression model used to discuss the impact of technology transfer on GIE may have endogenous problems (Zhou et al., 2020). Instrumental variable (IV) method is a common method to deal with the endogeneity of panel data (Lu et al., 2018). We use lag variables as a tool to deal with endogeneity problems. In Model 9, the Wald test shows that the original hypothesis of exogenous is accepted. At the same time, the results of the independent variables are also robust (see Table 6).

**Table 6:** Results of robustness tests.

**Variables**	**(7)**	**(8)**	**(9)**

	**Tobit**	**PCSE**	**IV-Tobit**
DTA	0.0215^**^	0.0484^**^	0.0466^***^
	(2.17)	(2.23)	(3.00)
FTI	0.0865	0.0658	0.0563
	(1.08)	(0.70)	(0.26)
ES	0.2789^***^	0.1797^***^	0.1752^***^
	(5.17)	(5.51)	(3.50)
ML	0.0032^***^	0.0064^***^	0.0065^***^
	(5.70)	(12.99)	(12.63)
FDI	0.0002	−0.0025^***^	−0.0027^**^
	(0.14)	(−3.80)	(−2.46)
HC	0.0358	0.0663^***^	0.0673^***^
	(1.34)	(13.27)	(5.65)
ER	0.0326^***^	0.0255^***^	0.0168
	(3.46)	(3.25)	(1.57)
ER^2^	−0.0013^***^	−0.0011^***^	−0.0006
	(−2.67)	(−2.66)	(−1.04)
MID	0.0786	0.0454^***^	0.0441^*^
	(1.41)	(5.51)	(1.87)
WEST	0.1630^***^	0.1472^***^	0.1481^***^
	(3.07)	(11.27)	(6.47)
Constant	0.1498^**^	−0.0251	0.0005
	(2.14)	(−0.49)	(0.01)
LR test	228.38^***^		
Wald test			0.5990
Observations	300	300	270

^***^p < 0.01, ^**^p < 0.05, ^*^p < 0.1.

## 5. Discussion

Some studies use the NDEA model to measure the innovation efficiency of China's HTI (Chen et al., 2018; Wang et al., 2020), but these studies do not consider environmental pollution in the innovation process of HTI. Differently from previous research, this paper uses the three-stage network DEA model to measure the GIE of China's HTI, because this deepens our understanding of the GIE in China's HTI.

DTA has significantly promoted GIE in Chinese HTI. This result is in accord with the findings of Liu et al. (2020), even though the two studies apply different measurement methods for dependent variables and independent variables (Liu et al., 2020). Under the development concept of green innovation, China actively supports enterprises to form strategic alliances with universities and research institutes for collaborative technological research. This enhances the green innovation capability of Chinese HTI; thus, DTA has a significant positive effect on the GIE of HTI.

The impact of FTI on the GIE of China's HTI is not significant. This result is different from the discovery made by Liu et al. (2020), who found that FTI hinders GIE in areas where HTI is highly developed. Although FTI will produce a technology spillover effect to some extent (Belitz and Molders, 2016). However, due to the lack of the technology absorptive capacity of most enterprises, it is difficult for China's HTI to obtain the corresponding economic and environmental benefits, so its impact on the GIE of HTI is not significant.

Existing research on the GIE of China's HTI generally regards green innovation as a “black box” (Li L. et al., 2018; Luo et al., 2019). According to the process perspective, green innovation in HTI is a three-stage system including R&D, commercialization, and diffusion. Therefore, we establish a three-stage NDEA model considering environmental pollution to measure GIE. This method provides an improved method for measuring the GIE of China's HTI.

The effectiveness of green innovation is affected by the source of technological knowledge, but most importantly by the combination of technological knowledge in the process of green innovation (Ben Arfi et al., 2018). However, there is little literature on the impact of various ways of technology transfer on GIE in China's HTI from a process perspective (Liu et al., 2020). We bring the two main ways of technology transfer, DTA and FTI, into a unified framework and discuss the impact of technology transfer on GIE from a process perspective. This research has deepened our understanding of the role of technology transfer in improving GIE in HTI.

## 6. Conclusion

Based on panel data from 30 provinces in China from 2011 to 2020, we used the three-stage NDEA model to evaluate the GIE of China's HTI and used the Tobit model to analyze the impact of DTA and FTI on the GIE of HTI. The results show that the average GIE of China's HTI is 0.7727. Except for four provinces, green innovation in most provinces is ineffective. DTA significantly promotes the improvement of GIE of China's HTI, while the impact of FTI on the GIE of HTI is not significant.

### 6.1. Implications for practice and policy

At present, the competition in high-tech field is increasingly fierce, and DTA has become an important approach to elevate GIE in Chinese HTI. In the process of actively promoting the construction of the national technology transfer system, China should pay more attention to improving its national technology trading network platform to provide information resources for high-tech enterprises to obtain appropriate domestic technologies. Moreover, it should actively support high-tech enterprises to build strategic alliances supporting industrial green innovation with universities and research institutions and should proceed green R&D and commercialization activities in line with market demand.

Although FTI has no significant positive impact on the GIE of HTI, we should not give up opening and technological cooperation. While actively introducing foreign advanced technology, it is necessary to enhance the absorptive capacity to realize the integration and utilization of foreign technological resources. In addition, an international technology transfer platform must be established to provide information services for the introduction of foreign technology. This can not only reduce the opportunity cost of introducing technology to high-tech enterprises, but also improve the applicability of imported technology to these enterprises.

### 6.2. Limitations and future research

According to the process perspective, we analyzed the influence of DTA and FTI on GIE in HTI under a unified framework and conducted an empirical test on the effect of these two types of technology transfer on efficiency improvements at each stage of green innovation. This study had two shortcomings, though. First, we introduced environmental pollution into the GIE analysis framework to explore the influence of technology transfer on GIE in HTI. However, because of the availability of data, we did not consider other undesirable output factors, such as wastewater and carbon dioxide emissions, when measuring the GIE. Second, Chinese HTI include pharmaceutical manufacturing, aviation equipment manufacturing, communication equipment manufacturing, and other sub-industries. Differences in the technological characteristics of these various sub-industries will affect the decision making about technology transfer. When we studied the relationship between technology transfer and GIE in HTI, we did not consider industry heterogeneity. These areas will be the focus of our next study.

## Data availability statement

The original contributions presented in the study are included in the article/supplementary material, further inquiries can be directed to the corresponding author.

## Author contributions

Methodology, software, validation, formal analysis, data curation, and writing—original draft preparation: SZ. Conceptualization, writing—review and editing, supervision, and project administration: FP. All authors have read and agreed to the published version of the manuscript.
